# Preparation of nickel/PA12 composite particles by defect-induced electroless plating for use in SLS processing

**DOI:** 10.1038/s41598-018-31716-8

**Published:** 2018-09-07

**Authors:** Chengmei Gui, Zhenming Chen, Chenguang Yao, Guisheng Yang

**Affiliations:** 1 School of Chemistry and Chemical Engineering, Hefei University of Technology, Hefei, Anhui 230009 China; 2 Guangxi Key Laboratory of Calcium Carbonate Resources Comprehensive Utilization, College of Materials and Environmental Engineering, Hezhou University, Hezhou, Guangxi 542899 China; 3 Shanghai Genius Advanced Materials Co., Ltd., Shanghai, 201109 China; 4 Hefei Genius Advanced Materials Co., Ltd., Hefei, Anhui 230009 China

## Abstract

In this work, Ni particles/PA12 powders (Ni/PA12) and graphite oxide (GO)-encapsulated Ni particles/PA12 powders (GO-Ni/PA12) composite powders were prepared by defect-included electroless plating technique, and its laser sintered behaviour was investigated. Results showed that a lot of defects could formed on the surface of CH_3_COOH etched PA12 powders. The defects would induce Ni and GO-Ni particles independently plated on the PA12 surface. Adding GO in the plating solution would facilitate the deposition of Ni particles, GO, and NiO on the PA 12 surface, but inhibit the growth and the crystallinity of the Ni particles. The SLS process involved the contact of PA12 powders, the formation of sintering neck, the growth of sintering neck and the formation of fused solid. Sintering process could facilitate the re-arrangement of Ni particles due to surface tension and the growth of sintering neck. The Ni particles had well wettability, and the interfaces between Ni particles and PA 12 were contacted soundly. The tensile strength and bending strength of the 10 W-sintered Ni/PA12 specimen were 50 MPa and 60 MPa. But SLS process caused the serious aggregation of GO-Ni particles due to higher concentration, activity and surface area of GO-Ni particles.

## Introduction

Nylon 12 (PA12) powder is most widely applied material in selective laser sintering (SLS), due to lower processing temperatures, lower sintering laser power and high accuracy of sintered part^1–5^. But the mechanical properties, especially impact strength, are lower than those of conventional compression molded part^5^.

At present, fillers, such as carbon nanotubes^6^, graphite oxide (GO)^5^, SiO_2_^7^ and metal particles^8^ have been widely used to reinforce the PA12 SLS parts. Those fillers can usually improve tensile strength, tensile modulus and impact strength. Conventional methods such as dissolution-precipitation^6^, melt mixing^1^, and solid state mixing^9^ have been widely used in preparation of filler-polymer composites. Yan *et al*.^8^ found that tensile strength, flexural strength, and flexural modulus of the Al-PA12 SLS parts were 110.4%, 162.4% and 222.3% higher than those of PA12 SLS parts, respectively. The dispersed state of composite filler and interfacial strength (filler-PA12) have significant effect on properties of final part. The lower dispersed state and interfacial strength difficult to achieve a stable complex system and transfer load effectively^2–8^.

It is well known that electroless plating is low cost process for surface modification on all kinds of particles, which can *in-situ* form composite particles on surface of substrate, with the primary adhesion and dispersion are very good^10–12^. However, the polymer surface is inert. Pretreatment can improve the interfacial adhesion between metal catalysts and polymer surface^13,14^. Song *et al*.^13^ reported a novel and facile activation for the electroless nickel plating of PS microspheres. Li *et al*.^14^ used the mixture of ethanol and water as the continuous phase, magnetic nickel layer was deposited onto the P(Py-PyCOOH) layer of the microspheres through an activation-electroless plating technology. They found compact and smooth metal coatings plated on the polymer surface. These techniques have to introduce activated sites on the polymer surface. The activated sites absorbed the catalytic particles via chelation. Dense reactive place was formed, which is the major causes of severely agglomerated plated powders. Control distribution of activated sites is the most effective method for obtaining uniform distribution of plated particles^13–17^. The preparation of uniform distribution of metal particles on PA12 powders surface by electroless plating technique is still not well studied.

When the plated composite powders subjected to selective laser sintering, laser directly contact with polymer and metal particles simultaneously^1–5^. The status of the metal particles in polymer melt and interactions between particles are the key factor for dispersed state of metal particles in polymer substrate. It is important to unveil the laser sintered behaviour about plated composite powders, which is especially important for understanding strengthening mechanism. The present work is mainly the preparation of Ni particles/PA12 powders (Ni/PA12) and GO-encapsulated Ni particles/PA12 powders (GO-Ni/PA12) composite powders by defect-included electroless plating technique, used in SLS molding technology, and research its laser sintered behaviour.

## Experimental Details

### Material

The commercial PA12 powder supplied by Evonik Industries AG (FORMIGAP110), with average particle size of about 20–100 μm (D90: 91 μm, D50: 55 μm, D10: 32 μm). The apparent density is 0.48 g/ml. The GO was bought from the Sixth Element Materials Technology Co., Ltd. (SE3122).

### Electroless plating process

The PA12 powders were immersed in acetic acid solution at 90 °C for 30 min. Then the PA12 powders were cleaned by deionized water. After that, the PA12 powders were immersed in electroless nickel plating or electroless composite plating bath.

A mixture of solution consisting of NiSO_4_·7H_2_O (5 mg/L) and Na_3_C_6_H_5_O_7_·2H_2_O (8 mg/L) was mixed at 333 K over a period of 1 h. The NH_4_Cl (18 mg/L) and NaH_2_PO_2_·H_2_O (15 mg/L) was then added. The mixture was left to stir for another 30 min. The plated solution was then obtained. GO dispersion (40 mg/L) was added in the plated solution and stirred well, and then the electroless composite plating bath was obtained. The bath temperature was 60 °C. The pH value was 9. The plated time was 100 min. The plated powders were cleaned by deionized water. Then filtered the plated powders through 250 mesh sieve.

### Selective laser sintering

The sintering experiments were carried out in an atmosphere of nitrogen gas using an HRPS-III SLS system (HUST). The SLS system was equipped with continuous wave CO_2_ laser (λ = 10.6 μm). The laser beam speed was 2000 mm/s, scan spacing was 0.1 mm, part bed temperature was 170 °C. A single-tier SLS parts were prepared for studying SLS mechanism of composite powders. The laser power were 3 W, 6 W and 10 W. To evaluate the composite powder, SLS parts were prepared in the laser sintering system. Limited to experimental conditions, milled mixtures of plated PA12 powders (5%) and pristine PA12 powders (95%) used to SLS materials.

### Characterization of the developed powders

Surface morphology of sample was observed by SEM (JEOL, JSM-5600LV). The chemical structure of the sample was measured by X-ray photoelectron spectroscopy (XPS, Shimazu, AXIS ULTRADLD, hν = 1486.6 eV). The thermal behavior of sample was studied by differential scanning calorimetry (DSC) and thermogravimetric analysis (TG, TA, Q2000). In this work, testing temperature was chosen from 50–750 °C, with an interval of 10 °C. The chemical structure of the sample was measured by X-ray diffraction (XRD) and X-ray photoelectron spectroscopy (XPS, Shimazu, AXIS ULTRADLD). The XRD pattern (15° to 60°) was equipped with a Cu Ka radiation (k = 0.154056 nm) source. XRD pattern (15° to 60°) was recorded on a Rigaku D/Max 2500 PC diffractometer equipped with a Cu Ka radiation (k = 0.154056 nm) source. The binding energy obtained in XPS analysis were corrected with the reference to C 1 s (284.6 eV). The surface area of powder was measured by N_2_ adsorption Brunner-Emmet-Teller method (Quantachrome, Autosorb-1-MP).

### Mechanical properties and SEM characterization of the laser sintering specimens

The tensile properties of the specimen was determined according to universal testing machines (Instron 33 R 4466, ASTM D638). A displacement control with a velocity of 2.54 mm/s was applied. The flexural properties were measured based on three-point bending test (790/MTS 810 Material Test System, ASTM D). The crosshead speed is 1.27 mm/min and the span is 2 inches. Each data point reported is an average of five repetitions. The microscopic morphologies of specimens were observed by SEM.

## Experimental Results and Discussion

**Figure 1:**
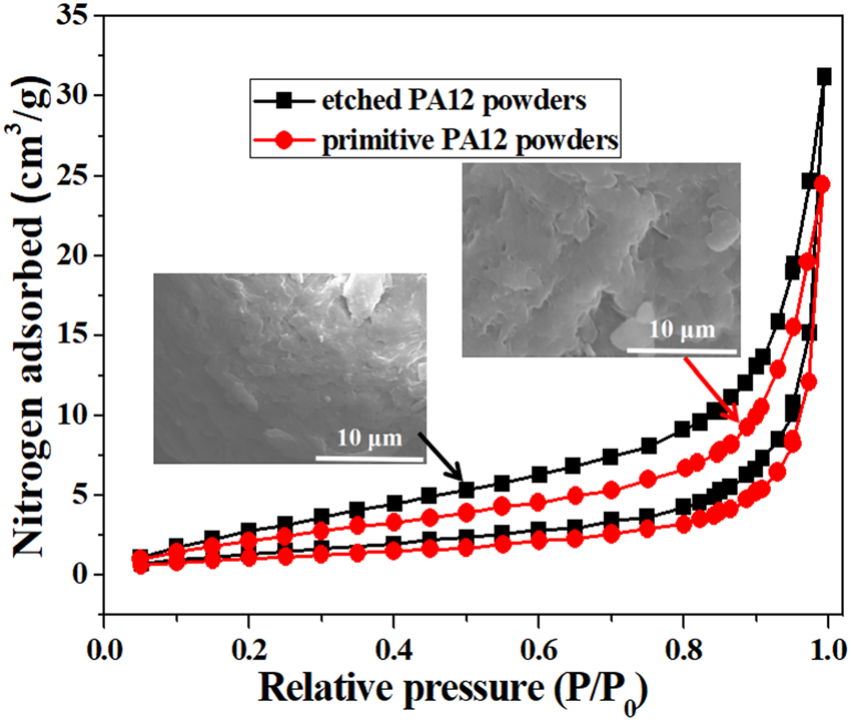
Relative pressure (P/Po) images of pristine PA12 powders and CH3COOH etched PA12 powders.

Figure 1 shows relative pressure (P/Po) of pristine PA12 powders and CH_3_COOH etched PA12 powders. The surface area of etched PA12 powders and pristine PA12 powders BET were 5.567 and 4.199 m^2^/g of N_2_ adsorption and desorption isotherms. It was found that the amount and size of hole on the surface of acetic acid etched PA12 powders were increased (as shown in the SEM images). The results indicated that a lot of defects could formed on the surface of acetic acid etched PA12 powders.

**Figure 2:**
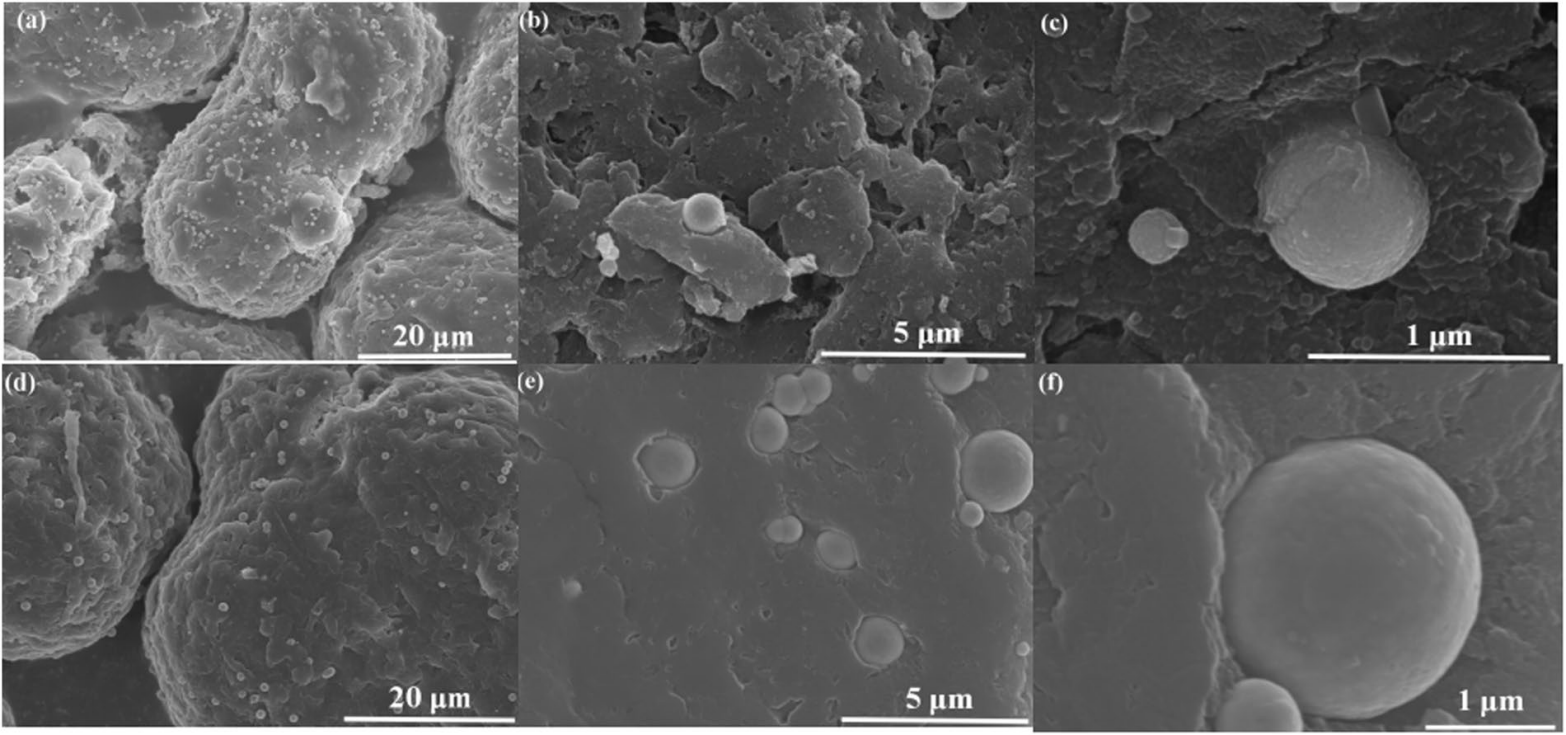
SEM images of GO-Ni/PA12 (**a–c**) and Ni/PA12 (**d–f**) composite powders.

Figure 2 shows SEM images of GO-Ni/PA12 (a–c) and Ni/PA12 (d–f) composite powders. As shown in Fig. 2, the Ni particles are deposited at surface defects. GO were coated on the surface of Ni particles, as shown in Fig. 2(c). It was indicated that Ni and GO-Ni particles could be prepare on the surface of etched PA12 powders after electroless plating. In addition, as shown in Fig. 2(a,d), the surface of GO-Ni/PA12 composite powders have more nickel particle than Ni/PA12 surface. The Ni and GO-Ni particles existed independently on PA12 surface. The average size of Ni particles in the Fig. 2(a) is about 430 nm, the one in the Fig. 2(d) is about 800 nm. The results indicated that adding GO in the plating solution would facilitate electroless nickel plating on the defects, but inhibit the growth of the Ni particles. The size of plated was 20–100 μm (D90: 92 μm, D50: 56 μm, D10: 35 μm). The effect of plating on particle size is not remarkably. The apparent density of plated particles increased to 0.66 g/ml.

**Figure 3:**
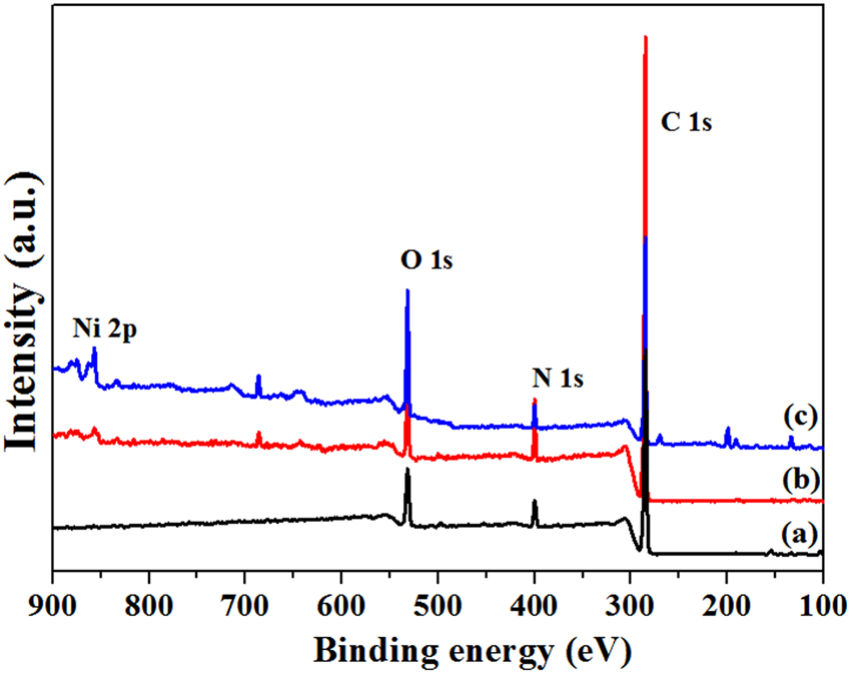
XPS spectra of acetic acid etched PA12 powders (**a**), Ni/PA12 (**b**) and GO-Ni/PA12 (**c**) composite powders.

**Figure 4:**
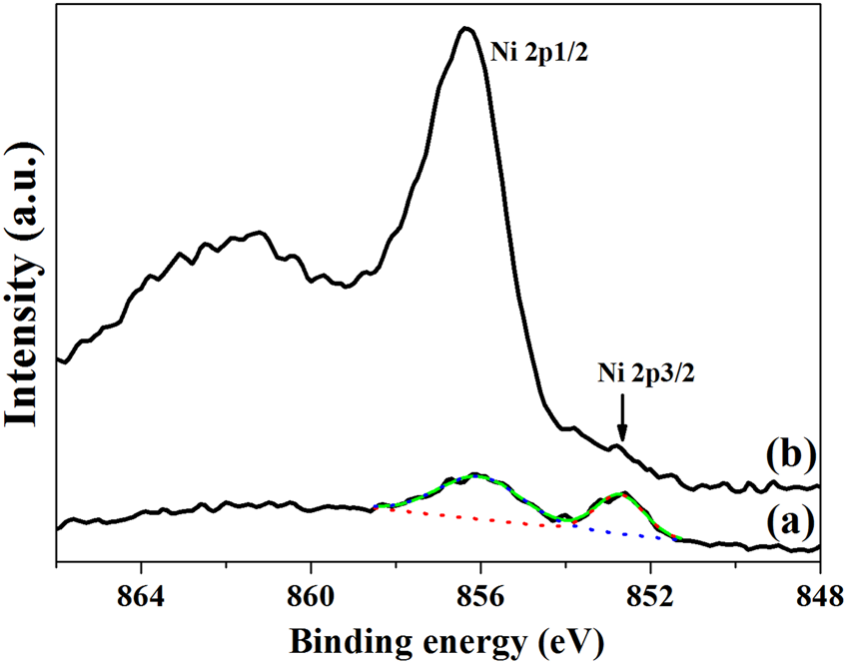
Ni XPS spectra of Ni/PA12 (**a**) and GO-Ni/PA12 (**b**) composite powders.

Figure 3 shows XPS spectra of acetic acid etched PA12 powders (a), Ni/PA12 (b) and GO-Ni/PA12 (c) composite powders. The peaks at about 286 eV, 398.8 eV, 534 eV, and 855 eV are due to the C 1 s, N 1 s,O 1 s, and Ni 2p, respectively. As shown in Fig. 3(b,c), Ni signal was detected in the spectra. It was indicated that nickel atoms were deposited on the surface of PA12 powders after electroless plating, nickel content of Ni/PA12 and GO-Ni/PA12 composite powders were 4% and 7%, respectively. It was indicated that the addition of GO in the plating solution would facilitate the deposition of nickel atoms on the surface of PA12 powders. Figure 4 shows Ni XPS spectra of Ni/PA12 (a) and GO-Ni/PA12 (b) composite powders. The Ni 2p peak was fitted into two chemical states, corresponding to Ni° (852.2 eV, Ni 2p_3/2_) and Ni^2+^ (856.6 eV Ni 2p_1/2_)^15,17^. For the purpose of quantitative investigation, we calculated the peak intensity ratio I_Ni 2p3/2_/I_Ni 2p1/2_ = 0.54 in Ni XPS spectra of Ni/PA12 composite powders, the other one is 0.08. The I_Ni 2p3/2_/I_Ni 2p1/2_ was weak in Ni XPS spectra of GO-Ni/PA12 composite powders, which indicated that most of the oxidized Ni species in the plated coating.

**Figure 5:**
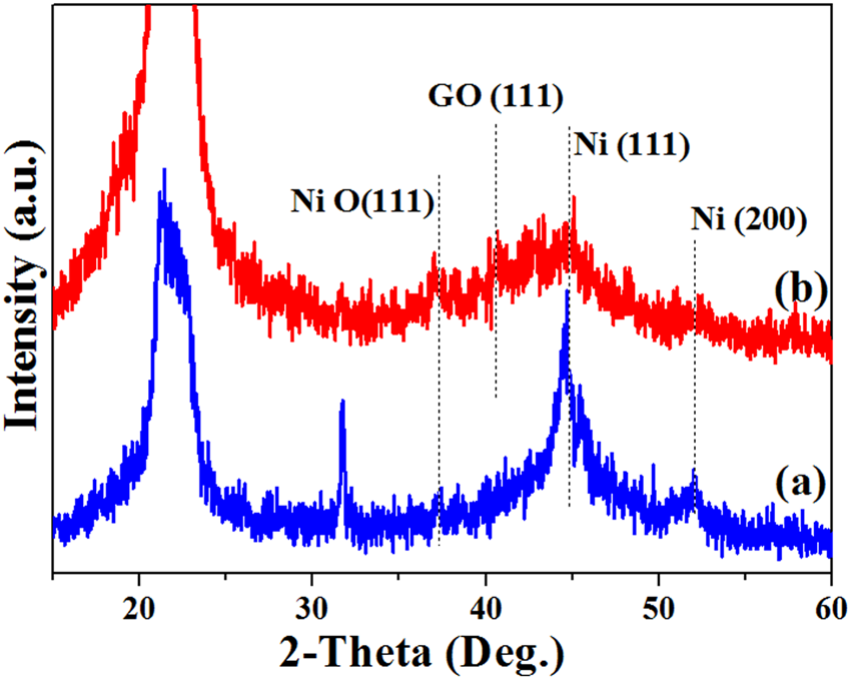
XRD patterns Ni/PA12 (**a**) and GO-Ni/PA12 (**b**) composite powders.

Figure 5 shows XRD patterns Ni/PA12 (a) and GO-Ni/PA12 (b) composite powders. The peaks at about 22°, 37°, 42°, 45° and 52° belong to PA12, NiO(111), GO(111), Ni(111) and Ni(200) diffraction peaks^15,17,18^. As shown in Fig. 5(a,b), it was observed that nickel crystallites was plated on the surface of PA12 powders. The (111) peak of full width at half maximum (FWHM) was narrower in the Fig. 5(a) than the other one. The results indicated that the adding GO in the plating solution would hinder the crystallinity of nickel-plated coatings and the formation of more larger grain size. In addition, as shown in Fig. 5(b), GO(111) signal and stronger NiO(111) signal (relative intensity) were detected in the spectrum. It was indicated that GO and better crystallization NiO were deposited on the PA 12 surface after electroless composite plating.

**Figure 6:**
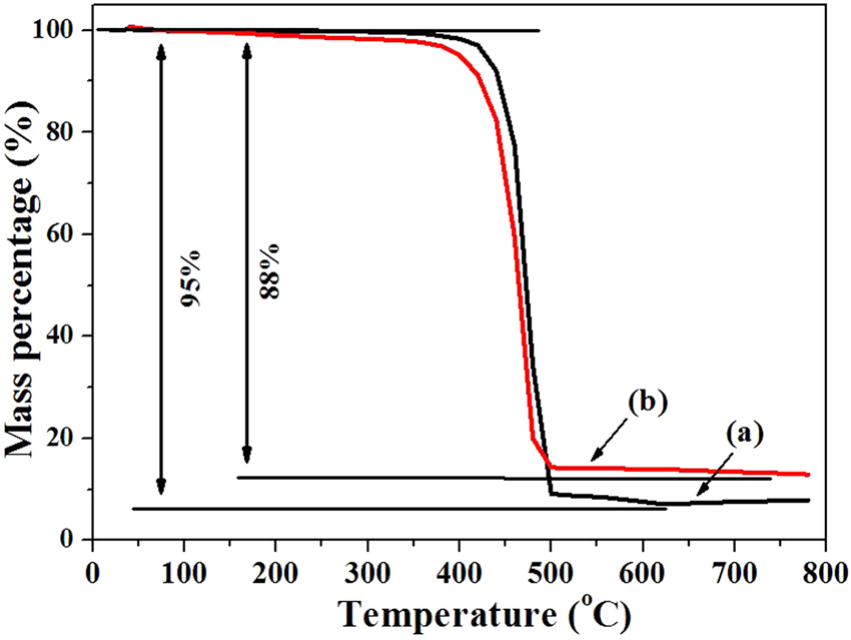
TG curves of Ni/PA12 (**a**) and GO-Ni/PA12 (**b**) composite powders.

Figure 6 shows TG curves of Ni/PA12 (a) and GO-Ni/PA12 (b) composite powders. As shown in Fig. 6(a), the deposited quantity (weight percent) of Ni/PA12 and GO-Ni/PA12 composite powders were 5% and 12%. The results indicated that added GO to the plating solution would facilitate electroless plating on the PA12 surface. In addition, melting temperature of composite powders is lower and crystallization temperature is higher than the ones of PA12 powders (DSC curves can be found in Figs S1 and S2 in Supplementary Data).

**Figure 7:**
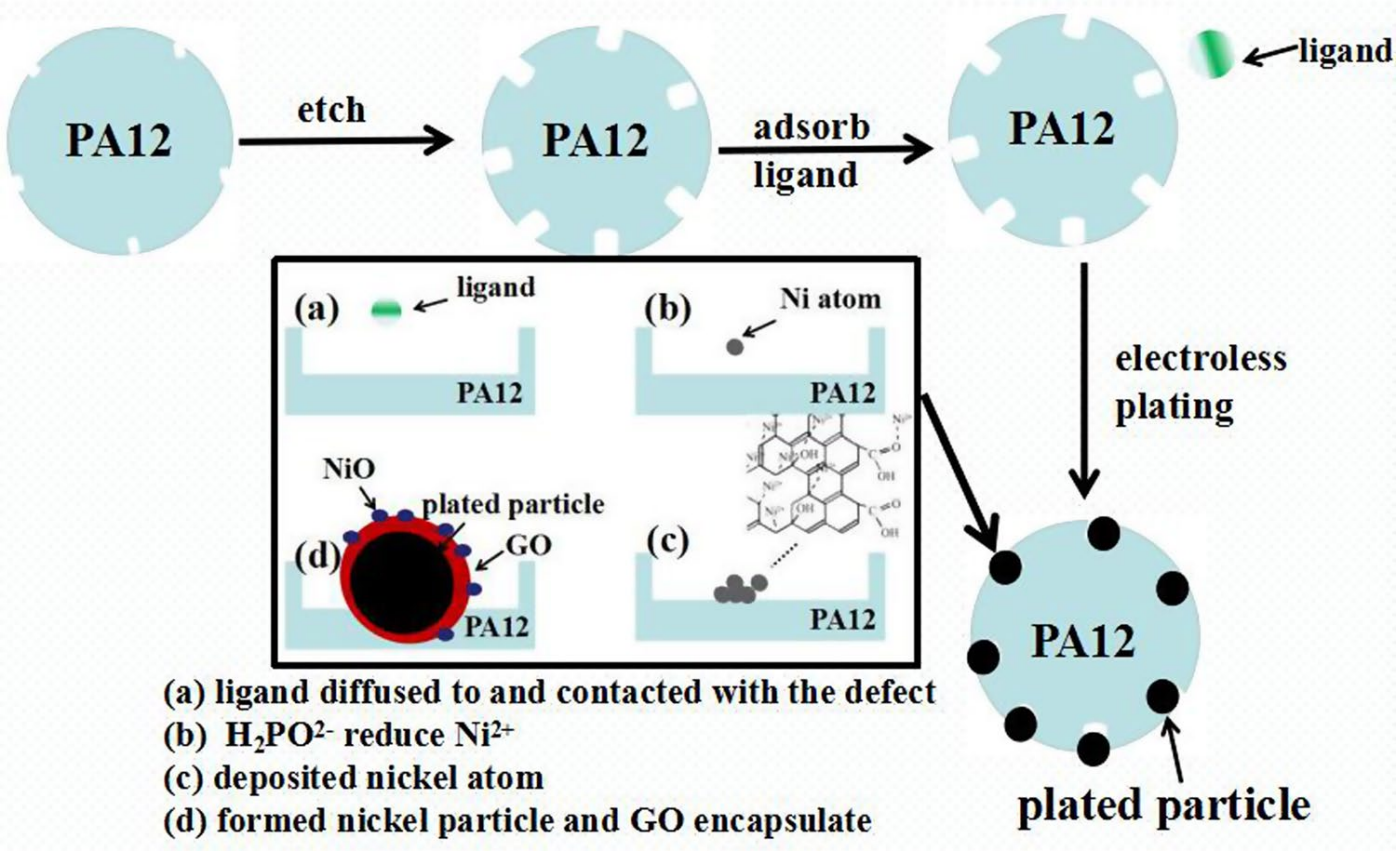
Schematic diagrams of the electroless plating procedure.

Figure 7 shows schematic diagrams of the electroless plating procedure. Ni^2+^ and C_6_H_5_O_7_^3+^ would form [Ni_4_(OH)_4_(C6H5O7)_3_]^5−^ ligand in the solution, sodium hypophosphite cannot reduce Ni^2+^ directly. When the [Ni_4_(OH)4(C6H5O7)_3_]^5−^ ligands diffused to the PA12 surface, the defects of its high chemical activity and capillary imbibition were expected to use as the scaffold for adsorption of the ligands, which would destroy the stability of ligands and reduce barrier of reaction^15–17^. The redox reaction occurred at the defects between a reducing agent ion (H_2_PO_2−_) and a plating ion (Ni^2+^), producing Ni atoms^10–12^. The reduced Ni can also be the medium, which cause the Ni^2+^ continuously deposited. The deposited nickel atoms aggregated and then formed Ni particles in the holes. The formation of plated coating involved some interface adhesion (such as diffusing, wetting, adhering) and chemical interaction^15–17^. In addition, defects of oxygen-containing functional groups and structure on GO surfaces could anchor Ni^2+^, and then formed NiO on the GO surface. As the same time, GO surfaces attracted many polar small molecules, for their high polarity. The plated Ni particles also have relatively high polarity, consequently, the GO were bonded by Ni particles, GO-Ni particles deposited in the holes. But the growth of Ni particles was stoped when its surface tightly coated with GO^19–21^. The GO dispersion contained surfactant which could improve the surface wettability of PA12 powder. It could facilitate the formation of good contact interface between solution and PA12 surface, which leaded to more reactive place forming. The results indicated that adding GO dispersion in the plating solution would facilitate electroless nickel plating, but inhibit the growth of the Ni particles.

**Figure 8:**
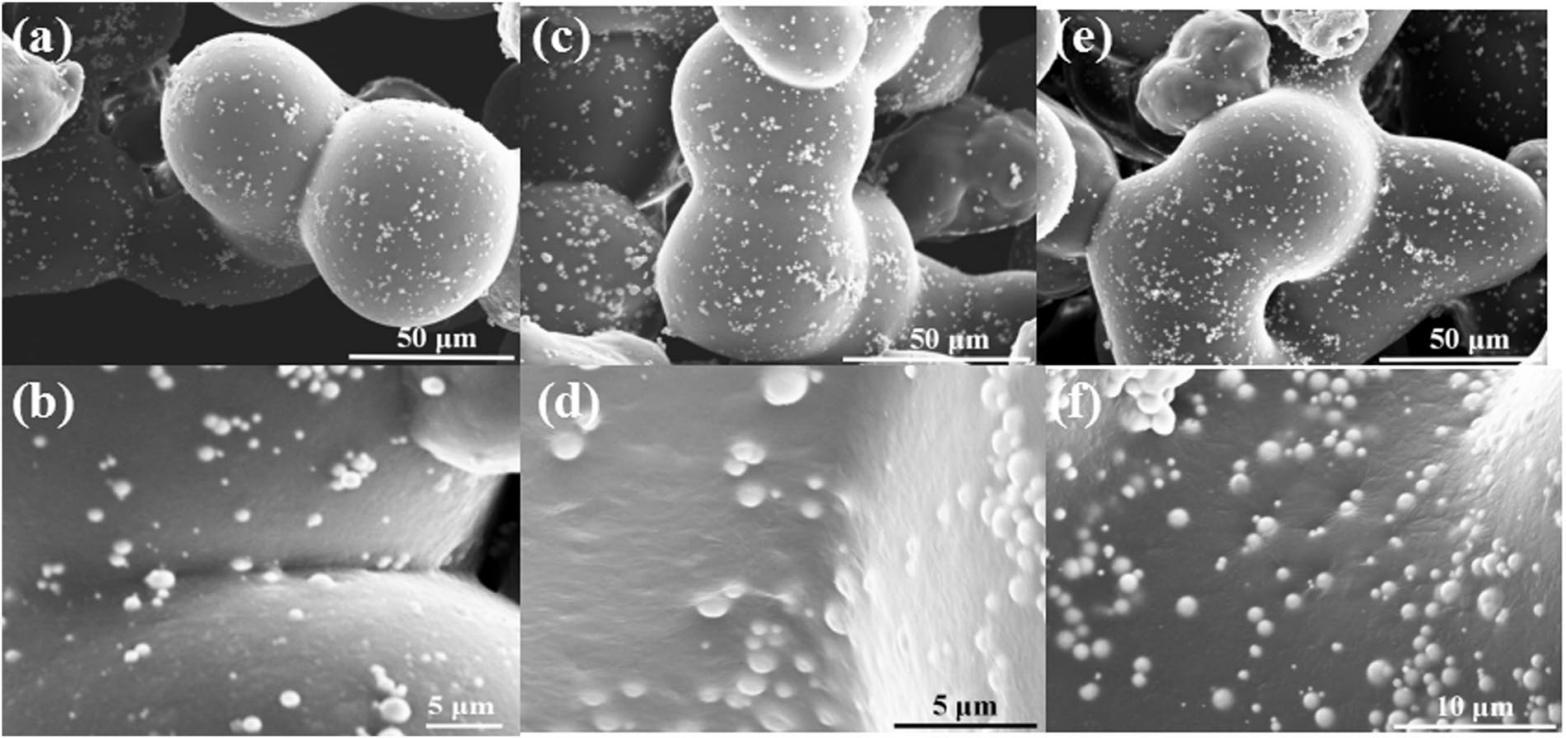
SEM images of Ni/PA12 composite powders subjected to selective laser sintering when the laser power were 3 W (**a,b**), 6 W (**c,d**) and 10 W (**e,f**).

Figure 8 shows SEM images of Ni/PA12 composite powders subjected to selective laser sintering when the laser power were 3 W (a,b), 6 W (c,d) and 10 W (e,f). As shown in Fig. 8(a,b), sintering necks were formed between the Ni/PA12 composite powders. Increasing the laser power, sintering necks were grew and then fused solid was formed between neighboring particles^1–5,7,8^, as shown in Fig. 8(c–f). In the process, Ni particles would move to the sintering necks and PA12 powders inside. It was also observed that there are no porosities, cracks and aggregates in it, and the PA12 powders can bind tightly to Ni particles. The results indicated that the surface of Ni particles had well wettability, and the interfaces between Ni particles and PA 12 were contacted soundly, thus, higher interfacial strength was obtained.

**Figure 9:**
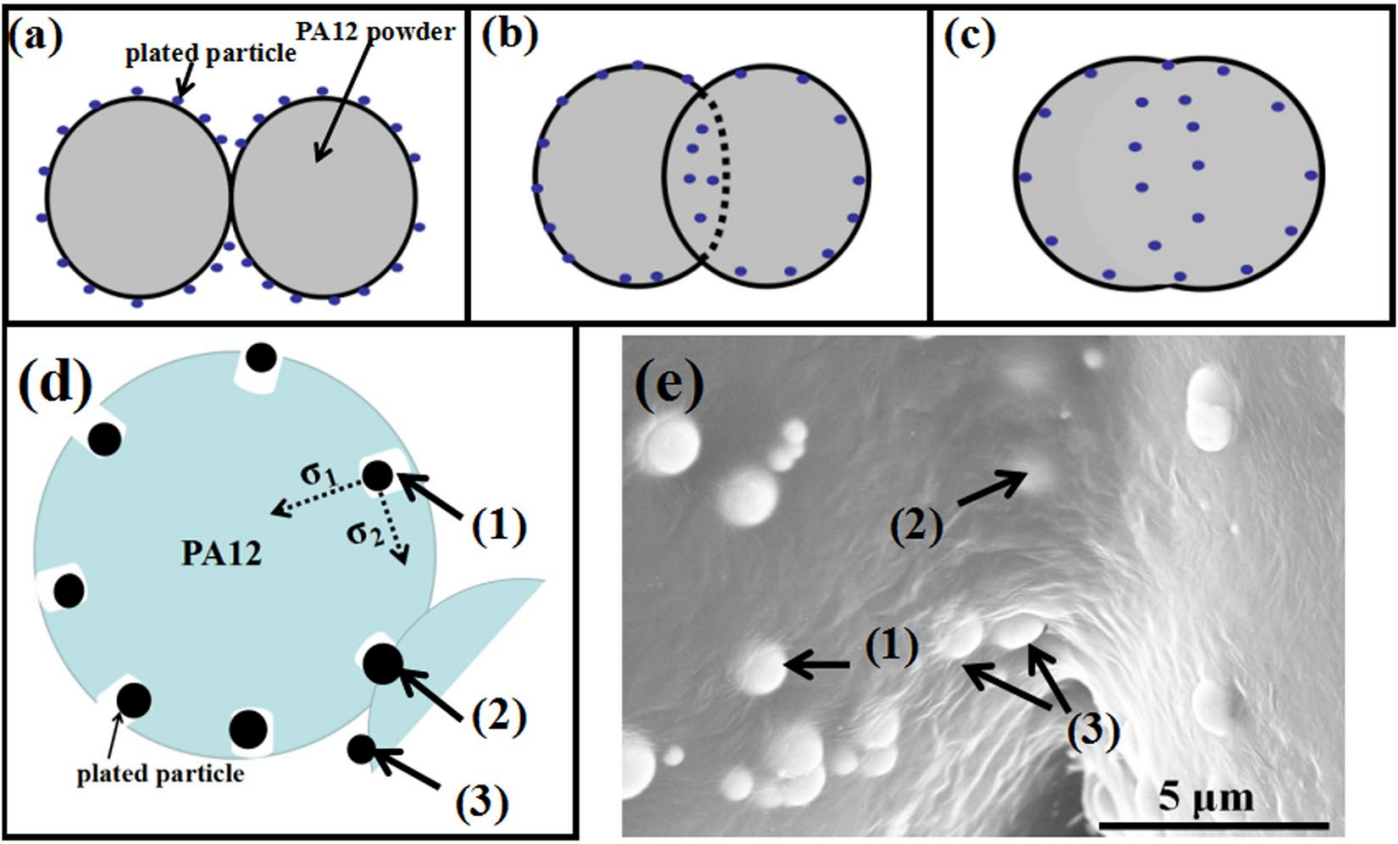
Schematic diagrams of SLS Ni/PA12 composite powders when the laser power were 3 W (**a**), 6 W (**b,d**) and 10 W (**c**), SEM images of 6 W-sintered Ni/PA12 composite powders (**e**).

Figure 9 shows schematic diagrams of SLS Ni/PA12 composite powders when the laser power were 3 W (a), 6 W (b) (d) and 10 W (c), SEM images of 6 W-sintered Ni/PA12 composite powders (e). The SLS process involves preheat the powder to 170 °C (a little lower than T_mp_), then a laser burns a series of lines into the dust, heating it to the T_mp_ of almost melting to form the object. Laser sintering is a thermal heating process, and thermal conductivity is a very important parameter to examine the performance of the materials during sintering process. It was reported that the Ni thermal conductivity is higher than that of PA12 powder. Increased powder thermal conductivity can improve the heat conduction during the laser-sintering process, resulting in denser and stronger parts. The laser sintering process of plated PA12 powders usually involves solid-phase sintering (before melting) and liquid-phase sintering^5,22–24^. Three stages could be distinguished during the solid-phase sintering according to kelvin theory: balling of particles, adhesion of particles, formation of sintering necks^22–26^. The porous PA12 powder was shaped by balling under the local laser high energy due to surface tension (indicated by σ_1_ in Fig. 9d). The Ni particles in the defects could get inside of PA12 powders under the σ_1_ action (indicated by (1) in Fig. 9). The contact interface between PA12 and Ni particles was good due to their similar polar. The PA 12 powders bonded together and then formed sintering neck, Ni particles get inside of two PA12 powders (indicated by (2) and (3) in Fig. 9d)^22–24^. With growing the sintering neck, more Ni particles get inside, as the same time, Ni particles on the PA12 surface was actuated by the other surface tension (indicated by σ_2_ in Fig. 9d), which was one of the main cause of growth of sintering neck. The σ_2_ would lead Ni particles migrate progressively to the sintering neck under lowest viscosity^22–25^, but the agglomeration of Ni particles would happen. Increasing the temperature to T_mp_, PA12 powder preform was viscous flow state. Within this range, the extinction of liquid phase accelerates the re-arrangement of the particles and enhances the densification by filling the clearance between PA12 powders, and then formed compact structure. Due to similar polarity and good compatibility, stable interfacial structure are formed^22–25^. In a word, the experiments and analysis results reflect the SLS process involved the contact of PA12 powders, the formation of sintering neck, the growth of sintering neck and the formation of fused solid.

**Figure 10:**
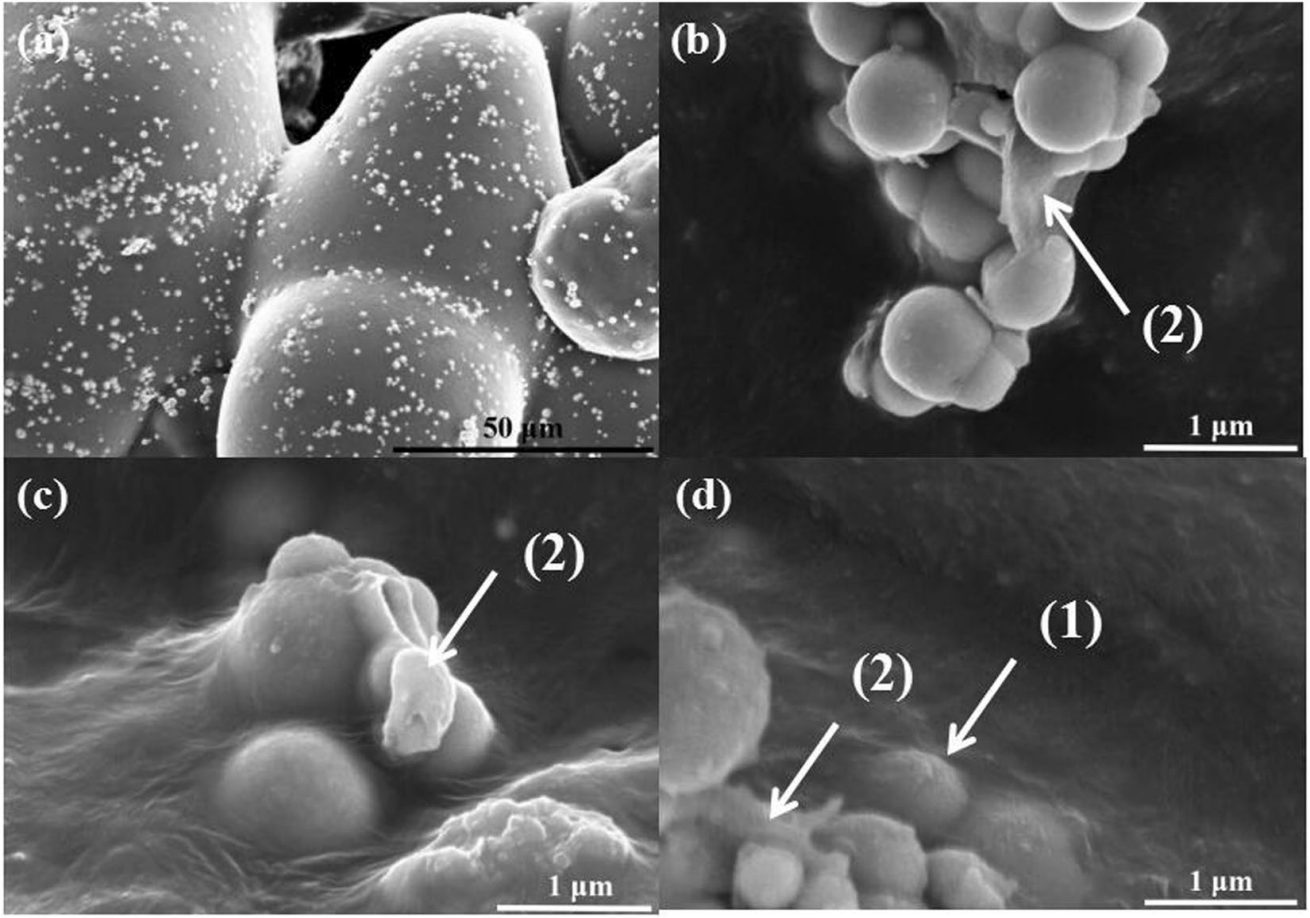
SEM images of GO-Ni/PA12 composite powders subjected to selective laser sintering when the laser power were 6 W.

Figure 10 shows SEM images of GO-Ni/PA12 composite powders subjected to selective laser sintering when the laser power was 6 W. As shown in Fig. 10, sintering necks were formed between the SLS Ni/PA12 composite powders, as shown in Fig. 10(a), the GO-Ni particles in the defects could get inside of PA12 powders (indicated by (1) in Fig. 10), GO sheets were coated on the surface of Ni particles (indicated by (2) in Fig. 9). But the SLS process caused the serious aggregation of GO-Ni particles, as shown in Fig. 10. There are two main reasons for the this. One is high concentration of GO-Ni particles on the PA 12 surface, the other is that GO-Ni particle possess high activity and surface area compared with Ni particle. The GO-Ni particles in the PA12 fluid likely attract each other due to higher attractiveness and lower particle spacing, and then agglomeration would happen.

Table 1 shows mechanical properties of SLS parts. From Table 1, it can be found that the tensile strength and bending strength of the Ni/PA12 specimen were lower than that of the PA12 specimen when laser power was 6 W. But when the laser power increased to 10 W, the tensile strength and bending strength of the Ni/PA12 specimen were higher than that of the 15 W-sintered pristine PA12 specimen. The tensile strength of the 10 W-sintered Ni/PA12 specimen was 50 MPa, the bending strength was 60 MPa. In addition, laser power has less influence on the strength of PA12 specimen than the one of Ni/PA12 specimen. The Ni particles would decrease contact area between the PA12 powder surface and laser, and increase the melt viscosity, which would decrease density of SLS part. Increasing laser power and decreasing rate are suggested to obtain high strength of SLS parts.

**Table 1:** Mechanical properties of SLS parts.

	laser Power (W)	Tensile strength (MPa)	Bending strength (MPa)	Elongation at break (%)
PA12 powder	6	34 ± 2.6	38 ± 4.2	26.8 ± 3.1
	10	42 ± 3.1	44 ± 1.9	20 ± 2.5
Ni/PA12 composite powder	6	21 ± 2.1	36 ± 2.5	21.1 ± 1.8
	10	50 ± 1.8	60 ± 3.5	10 ± 2.1

**Figure 11:**
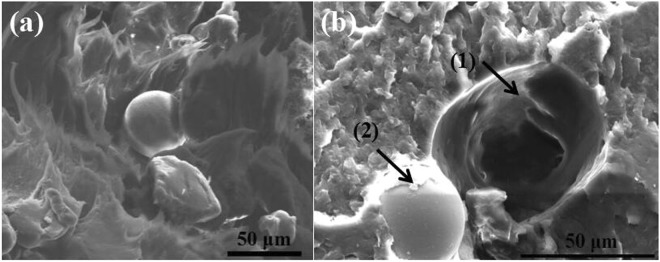
SEM images of fractured surface of the Ni/PA12 SLS specimens when the laser power were 6 W (**a**) and 10 W (**b**).

Figure 11 shows SEM images of fractured surface of the Ni/PA12 SLS specimens when the laser power were 6 W (a) and 10 W (b). From Fig. 11, it can be seen that the fractured surfaces of the 6 W-sintered Ni/PA12 specimen reveal a brittle behavior characterized by the large smooth area and ribbons, Ni particles separated from PA 12, indicating lower interfacial interaction between the Ni particles and the PA12 matrix, less energy required to fracture the specimens. The 10 W-sintered Ni/PA12 specimen has rougher fractured surface features (see Fig. 11(b)) such as shear yielding and crack pinning. In addition, PA12 partial covered on surface of Ni particle (see Fig. 11(2)) and the remaining hole (particle pullout) was deformed (see Fig. 11(1)). As more energy was required to form the deformation strain of specimens for the creation of these features, therefore the higher impact strength for the 10 W-sintered Ni/PA12 specimen was obtained. Particle reinforcement, crack deflection and particle pullout are the main toughening mechanisms.

## Conclusion

In this work, Ni/PA12 and GO-Ni/PA12 composite powders were prepared by defect-included electroless plating technique, and its laser sintered behaviour was investigated. Results showed that a lot of defects could formed on the surface of acetic acid etched PA12 powders, Ni/PA12 and GO-Ni/PA12 composite powders could be plate on the PA12 surface. Adding GO in the plating solution would facilitate the deposition of Ni particles, GO and better crystallization NiO on the PA 12 surface, but inhibit the growth of the Ni particles and the crystallinity of nickel-plated coatings. The deposited quantity of Ni/PA12 and GO-Ni/PA12 composite powders were 5% and 12%, the average size of Ni particles were about 800 nm and 430 nm. The SLS process involved the contact of PA12 powders, the formation of sintering neck, the growth of sintering neck and the formation of fused solid. Sintering process could facilitate the re-arrangement of Ni particles and get inside the SLS fused solid. The surface of Ni particles had well wettability, and the interfaces between Ni particles and PA 12 were contacted soundly. But SLS process caused the serious aggregation of GO-Ni particles. The tensile strength and bending strength of the 10 W-sintered Ni/PA12 specimen were 50 MPa and 60 MPa.

## Electronic supplementary material

Supplementary Material
