# Supplementary material for: Preparation of nickel/PA12 composite particles by defect-induced electroless plating for use in SLS processing
Source: Sci Rep. 2018 Sep 7;8:13407. doi: 10.1038/s41598-018-31716-8 (PMC6128841; doi:10.1038/s41598-018-31716-8)
Supplement: Supplementary file 1 — Supplementary Material [file 41598_2018_31716_MOESM1_ESM.doc]

**Preparation of nickel/PA12 composite particles by defect-induced electroless plating for**

**use in SLS processing**

*Chengmei Gui^1^**, Zhenming Chen^2^, Chenguang Yao^4^ and Guisheng Yang^1,3,4^**[[1]](#footnote-2)

*1 School of Chemistry and Chemical Engineering, Hefei University of Technology, Hefei, Anhui 230009, China*

*2 Guangxi Key Laboratory of Calcium Carbonate Resources Comprehensive Utilization, College of Materials and Environmental Engineering, Hezhou University, Hezhou, Guangxi 542899, China*

*3 Shanghai Genius Advanced Materials Co., Ltd., Shanghai 201109, China*

*4 Hefei Genius Advanced Materials Co., Ltd., Hefei, Anhui 230009,China*


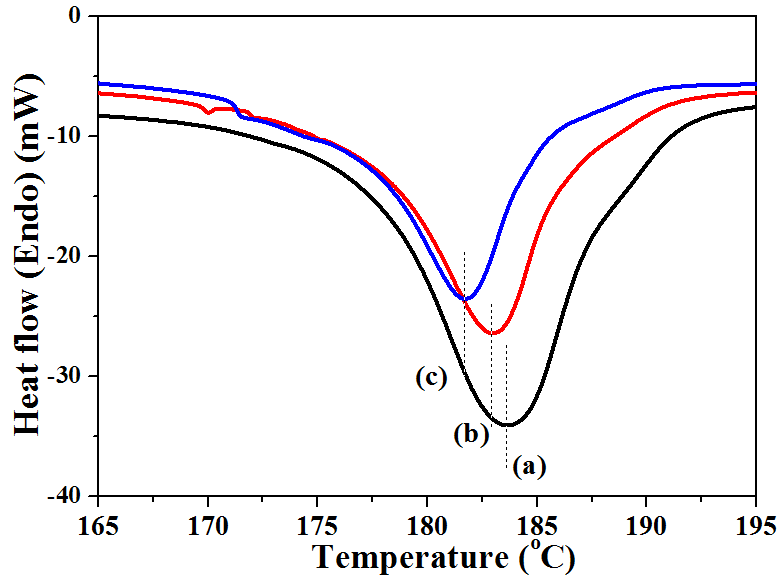


Fig.1 Heating DSC (a) curves of acetic acid etched PA12 powders (a), Ni/PA12 (b) and GO-Ni/PA12 (c) composite powders


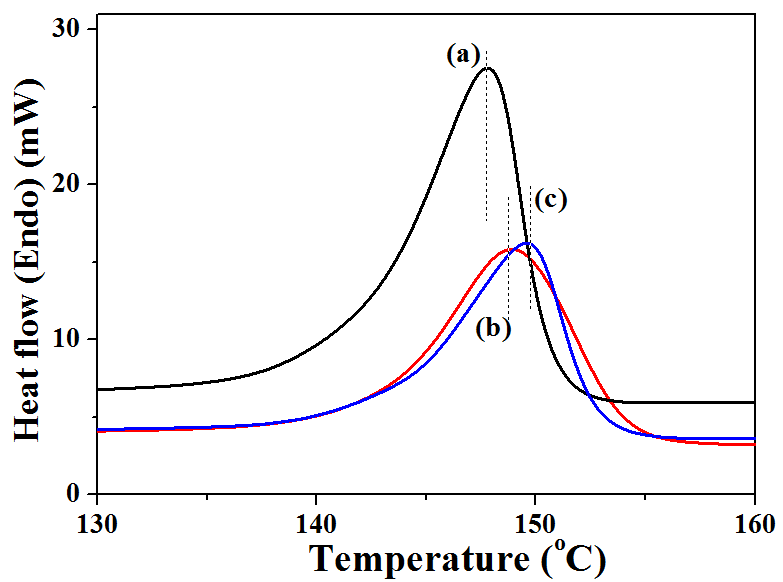


Fig.2 Cooling DSC curves of acetic acid etched PA12 powders (a), Ni/PA12 (b) and GO-Ni/PA12 (c) composite powders

Fig.S1 and Fig.S2 shows heating and cooling DSC curves of acetic acid etched PA12 powders (a), Ni/PA12 (b) and GO-Ni/PA12 (c) composite powders. The melting temperature (T_mp_) and crystallization temperature (T_cp_) of acetic acid etched PA12 powders were 184 ^o^C and 147 ^o^C, the ones of Ni/PA12 composite powders were 183 ^o^C and 148 ^o^C, the ones of GO-Ni/PA12 composite powders were 181 ^o^C and 150 ^o^C. The T_mp_ of plated powders lower than the one of etched PA12 powders, which could be explained by the remarkable higher thermal conductivity of plated coating in the holes. Moreover, the T_cp_ of plated powders was higher than that of pristine PA12 powders. The increase in the T_cp_ of PA 12 may be attributed to the heterogeneous nucleation effect of the plated particles, which makes PA 12 crystal grow more perfectly. In addition, T_mp_ of GO-Ni/PA12 composite powders is lower and T_cp_ is higher than the ones of Ni/PA12 composite powders due to more number of plated particles on PA12 surface.

1. ^*^ Corresponding author. G.S. Yang

   E-mail address: ygs@geniuscn.com [↑](#footnote-ref-2)
